# Novel synthesis method of multi walled carbon nanotube-silica Janus nanostructures

**DOI:** 10.1038/s41598-025-03164-8

**Published:** 2025-06-04

**Authors:** Hamed Namdar, Mehrdad Manteghian, Arezou Jafari, Masoud Riazi

**Affiliations:** 1 Department of Petroleum Engineering, Faculty of Chemical Engineering, Tarbiat Modares University, Tehran, Iran; 2 School of Mining and Geosciences, Nazarbayev University, Kabanbay Batyr 53, Astana, Kazakhstan

**Keywords:** Janus, Nanoparticle, Synthesis, MWCNT, Silica, Hydrophilicity, Oleophilicity, Synthesis and processing, Petrology

## Abstract

Nowadays, Janus nanoparticles have received a lot of attention in high-tech processes due to their special ability of simultaneous hydrophilicity and oleophilicity. In this study, a new synthesis method for Janus nanostructure using multi-walled carbon nanotube (MWCNT) and silica nanoparticle is proposed. FTIR, TEM, EDX, XRD and TGA characterization tests were performed to check the properties of synthesized nanostructure. The simultaneous hydrophilicity and oleophilicity properties of the synthesized nanostructure was investigated by examining its placement at the interface of water and various organic materials (engine oil, chloroform and crude oil). Also, effect of weight ratios of nanotubes to silica (10, 20, 30, 40 and 50%) on the degree of hydrophilicity and oleophilicity were investigated. The results of FTIR, XRD, EDX and TGA characterization tests effectively show the formation of nanotube-silica hybrid. Also, the FTIR test results confirm the hydrophilicity and oleophilicity of the synthesized nanostructure according to the absorption spectrum of carboxylic acid functional groups (hydrophilicity property) and alkane and alkene bonds related to oleic acid (oleophilicity property). In addition, TEM images clearly show the bonding of silica nanoparticle on the structure of MWCNTs. The average size of the synthesized crystal was estimated to be about 13 nm using XRD results, while with the help of TEM image analysis, the average particle size was estimated to be 14 nm. The physical presence of the synthesized nanostructure at the interface of water and various organic materials is a good confirmation of the dual nature of the synthesized nanostructure. The finer, more uniform and the largest amount of emulsion was formed at the nanotube to silica ratio of 20%, therefore Janus nanostructure has a better performance in this ratio.

## Introduction

Janus nanoparticle is a special type of nanoparticles (NPs) that have two surfaces with different physical and chemical properties. This type of unique structure of Janus particles allows two different types of chemical substances to form on one particle. The simplest form of Janus nanoparticle can be obtained by dividing the nanoparticle into two separate parts, each of which is made of a different material or covered with a different functional group^1^. For example, a Janus nanoparticle may be made of one surface with a hydrophilic functional group and another surface with an oleophilic functional group. This phenomenon gives to nanoparticle unique properties that depend on their asymmetric structure^2^. Cho and Lee reported in 1985 about a particle with the properties of Janus particles. But they still didn’t use the Janus term for the particles which they made^3^. The Janus term was used for the first time in the writings of Casagrande et al. (1989) to explain spherical glass particles with one hydrophilic end and the other hydrophobic end^4^. A few years later, Binks and Fletcher (2001) focused on the wettability of Janus spherical particles in the presence of water and oil. They introduced Janus particles as asymmetric materials that have two hydrophilic and oleophilic surfaces, while symmetrical materials have only one type of wettability^5^.

The synthesis of Janus nanoparticle is crucial. Janus nanoparticle can be designed for targeted applications in multiple domains, such as catalysis, pharmaceutical delivery, and environmental cleanup. Their capacity to combine contrasting characteristics, such as hydrophobic and hydrophilic properties, enables them to operate efficiently in a variety of settings and functions^6–8^. The dual properties of Janus nanoparticle enhance their performance in applications like drug delivery systems, allowing them to transport both hydrophilic and hydrophobic drugs concurrently. This ability can result in improved therapeutic outcomes by increasing target affinity and regulating release profiles^8,9^. The distinctive optical characteristics of Janus nanoparticle render them well-suited for imaging applications, facilitating more accurate diagnostics^7^. Janus nanoparticle function as efficient catalysts owing to their adjustable surface characteristics, which can be tailored to improve reaction outcomes. They possess the ability to stabilize emulsions and accelerate reaction rates in biphasic systems, thereby proving their significance in chemical synthesis methodologies^6,9^. In environmental contexts, Janus nanoparticle can aid in the degradation of pollutants or improve oil recovery methods, demonstrating their effectiveness in tackling ecological issues^9,10^. Janus nanoparticle serve as compatibilizers in polymer blends, enhancing the stability of immiscible mixtures. This function is vital in manufacturing processes where the compatibility of materials is crucial for maintaining product integrity^7^. The capacity to develop functionalized surfaces with customized characteristics paves the way for advancements in coatings and surface treatments across multiple sectors^6,9^. since the production of Janus nanoparticle is crucial because of their multifunctional properties, which serve a diverse array of applications spanning from biomedical sectors to industrial processes, some common methods of Janus nanoparticle synthesis will be described below.

### Synthesis methods of Janus particles

In order to synthesize Janus particles, it is necessary to put two surfaces of particles together in very small sizes, which each of these surfaces has completely different properties from the other surface. Undoubtedly, the production of these materials in the field scale will cost a lot, but since about 10 years ago, methods have been proposed for the easier production of these materials in the field scale. In this section, 3 of these methods are mentioned that have the most application and performance^11^.

#### Masking method

**Fig. 1:**
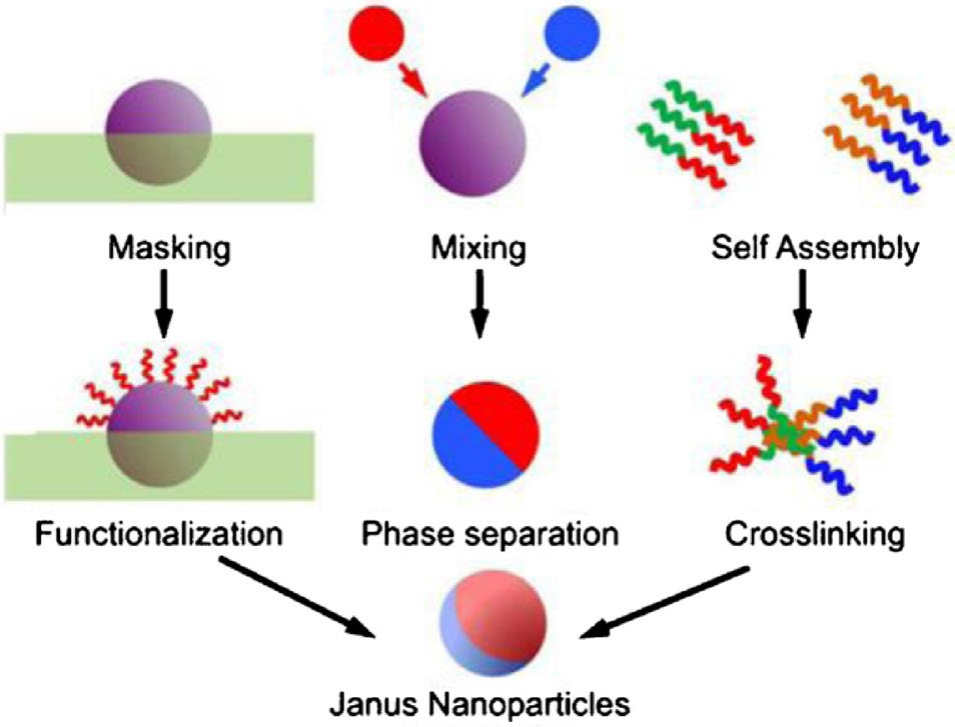
Schematic of the three methods of masking, phase separation and self-assembly for the synthesis of Janus nanoparticle^12^.

In the liquid-gas method, a surface of nanoparticle is placed in the liquid and the other surface is placed in presence of the vapor of another substance so that a Janus particle produced from the vapor settling on that surface (Fig. 1). Another method is to keep nanoparticle suspended in the boundary between two immiscible liquid-liquid and liquid-solid phases^12^.

In the liquid-liquid contact surface method, an emulsion of water and oil was made and iron nanoparticle were added to it. Iron nanoparticle gather on the contact surface of water and oil mixture and form an emulsion clot Then silver nitrate is added to the solution and the result is the deposition of silver nanoparticle on iron nanoparticle^13^.

#### Phase separation method

In this method, two incompatible substances are mixed together on the surface of a nanoparticle^14^. These two incompatible substances can be mineral substances or organic substances (Fig. 1). The use of organic materials to make Janus particles was developed by Yoshida et al. (2009). They made Janus particles, one hemisphere of which was made of human cells, while the other side had nothing to do with human cells^15^.

#### Self-assembly method

The synthesis of Janus particles by self-assembly method (Fig. 1) was presented for the first time in 2001 by Erhardt et al., they made a triple polymer block of polymethyl acrylate, polystyrene and light-weight polybutadiene. Polymethyl acrylate and polystyrene form alternating layers where polybutadiene is in the form of small spheres between these layers, then these polymers are chemically bonded together and after the bonding chemically, this triple polymer is dissolved in a solution of tetrahydrofuran. From this method, Janus particles are obtained, with polymethyl acrylate on one side, polystyrene on the other side, and polybutadiene in the center. With this method, it is possible to make spherical, cylindrical, plate, or even ribbon Janus particles^16^. In order to provide a proper perspective, the common methods of synthesis of Janus nanoparticle are briefly presented in Table 1. Table 1 shows the usual synthesis methods, compositions, particle size and morphology of a number of Janus structures.

**Table 1:** Common synthesis methods, compositions, particle size and shape of Janus particles.

Synthesis method	Composition	Shape	Particle size	References
Masking	SiO_2_-Au		Nano	^17^
	Ag-MoS_2_		Nano	^18^
	ZnO-PS		Micro	^19^
Phase separation	Al_2_O_3_-SiO_2_		Nano	^20^
	PtBA/PS		Nano	^21^
	PLLA/PS		Nano	^22^
Self-assembly	PS-PI-PMMA		Nano	^23^
	PS-b-P4VP		Nano	^24^
	PS-b-P_4_VP		Nano	^25^

Currently, a large collection of Janus particles with different methods, sizes and shapes have been made, which are used in textile industries, making electronic devices, stabilizing emulsions, magnetic imaging, enhanced oil recovery, etc. But Janus hybrid nanoparticle of nanotube-silica has not been synthesized yet. MWCNT and silica are two prevalent types of nanomaterials with high surface area, porosity, and surface functionalities. The reasons for paying attention to these hybrid nanostructures in the formation of Janus nanostructure and Pickering emulsions are the following:

- The possibility of creating a hydrophilic/hydrophobic structure^26^.
- The possibility of changing the amount of hydrophilicity and hydrophobicity of particles by changing the percentage of structural components^27^.
- Very suitable capabilities of MWCNT and low cost of silica.

Carbon-silica hybrid nanostructure consists of two different structures, one of which can be hydrophilic and the other hydrophobic. Among the carbon structures, we can refer to various nanotube structures, including single walled carbon nanotube or MWCNT and functionalized or open-ended nanotube structures, which due to their shape and structural properties, as well as the possibility of functionalization can have more applications in making Janus hybrids. Nanotubes are widely produced by chemical vapor deposition, but the grown nanotubes are often accompanied by the production of amorphous carbon and mixing with metal particles^28^. Achieving the desired properties in composites containing nanotubes depends on the homogeneous dispersion of nanotubes in the matrix^29^. One of the suitable methods to increase the interaction between the matrix and the nanotubes and the homogeneous dispersion of the nanotubes in the matrix is to create functional groups on the surface of the nanotubes^30^. Nanotube is inherently a hydrophobic material, of course, this material is not hydrophilic either, but its properties can be changed by partial oxidation and also by placing different functional groups on the structure of nanotubes^31^. For example, it is possible to add hydrophilic properties to nanotubes by placing carboxylic functional groups on the structure^32^. The intensity of functionalization of nanotubes can be controlled by controlling the reaction time and the concentration of reactive acid. The concentration of functional groups on the nanotube structure is possible from 4% by weight to higher percentages. Functional groups can be placed on the structure and in the opened parts of the nanotube during the opening of the closed end of the nanotube^33^.

In addition, the surface of unmodified nanotubes has many hydrophobic sites. One of the methods of functionalizing nanotubes is surface oxidation. The method of oxidizing the surface is to create functional groups using chemical methods. So far, many researchers have used this method to improve the dispersion of materials in the desired environment. Achieving the desired properties in composites containing nanotubes depends on the homogeneous dispersion of nanotubes in the matrix^29^. One of the suitable methods to increase the interaction between the matrix and the nanotubes and the homogeneous groups on the structure dispersion of the nanotubes in the matrix is to create functional groups on the surface of the nanotubes^30^. Functionalization is a chemical process based on which the desired functional groups (OH, C= O and COOH) are created on the surface of the material^32^.

Silica is one of the metal oxide compounds known to stabilize Pickering emulsions^34^. Nanotubes and silica are used in Janus nano hybrid because due to the difference in the structure and properties of these two materials, they can be given hydrophilic and hydrophobic properties, which are very suitable for forming suitable materials for creating Pickering emulsions.

Therefore, in this study, this type of Janus nanoparticle is synthesized in a new way and then, FTIR, TEM and XRD characterization tests were performed on it, in order to check the synthesized nanostructure. Also, in order to investigate the hydrophilicity and oleophilicity properties of the synthesized nanostructure, its placement at the interface of water and engine oil, water and chloroform, and water and crude oil was investigated. On the other hand, considering that the weight ratio of nanotubes to silica can play an important role in the degree of hydrophilicity and oleophilicity of the nanostructure, therefore, the synthesized nanostructures investigated in various different weight ratios of nanotubes to silica and the amount of emulsification and the size of the formed emulsions were investigated.

## Experimental

### Materials

The characteristics of the materials used in this study are shown in Table 2. The used crude oil belongs to one of the fields in the west of Iran, which has an API 18.9° and its viscosity is 678.96 centipoises at standard conditions.

**Table 2:** Specifications of the materials used in the study.

Material	Company	Application
Multi walled carbon nanotube	US research nanomaterials	Synthesis of Janus nanostructure
Nitric acid	Merck	Functionalization of carbon nanotubes
Sulfuric acid	Merck	Functionalization of carbon nanotubes
Silica nanoparticle	US research nanomaterials	Synthesis of Janus nanostructure
Paraffin wax	Merck	Surface modification of silica nanoparticle
Oleic acid	Merck	Surface modification of silica nanoparticle
Chloroform	Merck	Dissolving paraffin
Engine oil	One of the Iranian oil companies	Investigating the dual nature of Janus nanoparticle
Crude oil	One of the Iranian western oil reservoirs	Investigating the dual nature of Janus nanoparticle
Ammonia solution	Merck	Surface modification of silica nanoparticle
Deionized water	Deionizer-FNR-12	Synthesis of Janus nanostructure and washing

### Methods

**Fig. 2:**
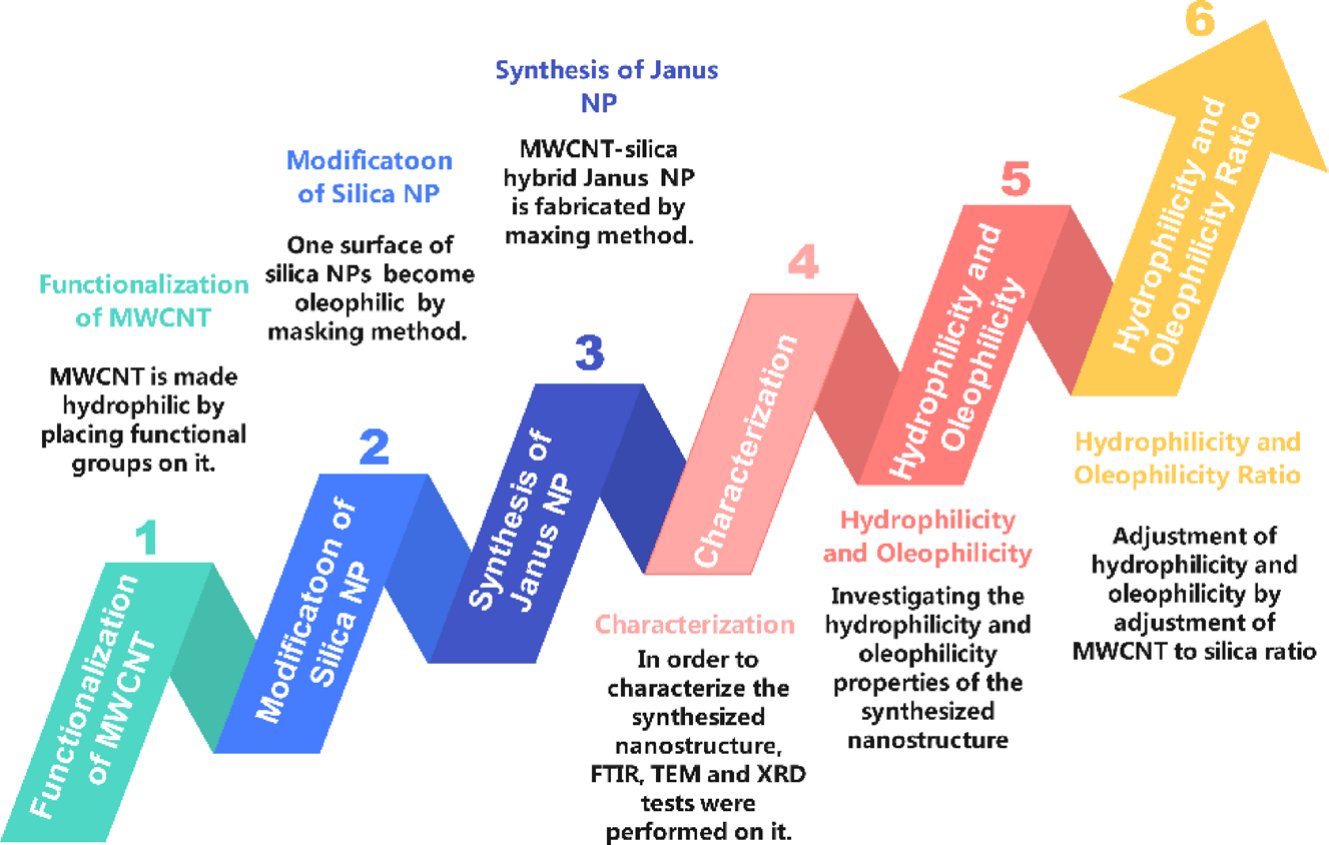
Steps of the study method.

With these explanations, the new method used for the synthesis of MWCNT-silica Janus nanostructure will be discussed in following. Figure 2 shows the steps of the study method.

#### Functionalization of MWCNT

**Fig. 3:**
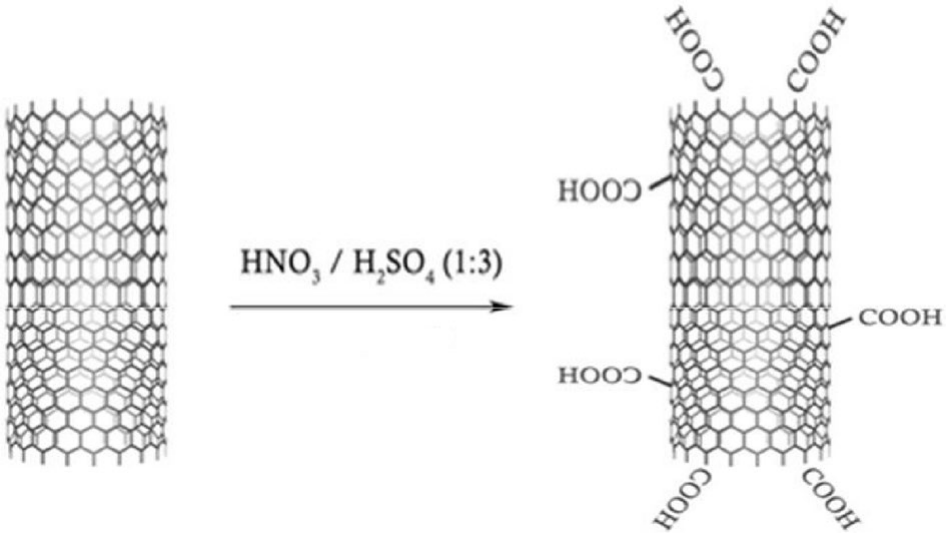
Schematic of chemical functionalization of MWCNT.

The surface of unmodified nanotubes has many hydrophobic sites. One of the methods used to make nanotubes hydrophilic is surface oxidation. The method of oxidizing the surface is to create functional groups using chemical methods. Figure 3 shows a schematic of chemically functionalized nanotubes whose method is explained in the following.

**Fig. 4:**
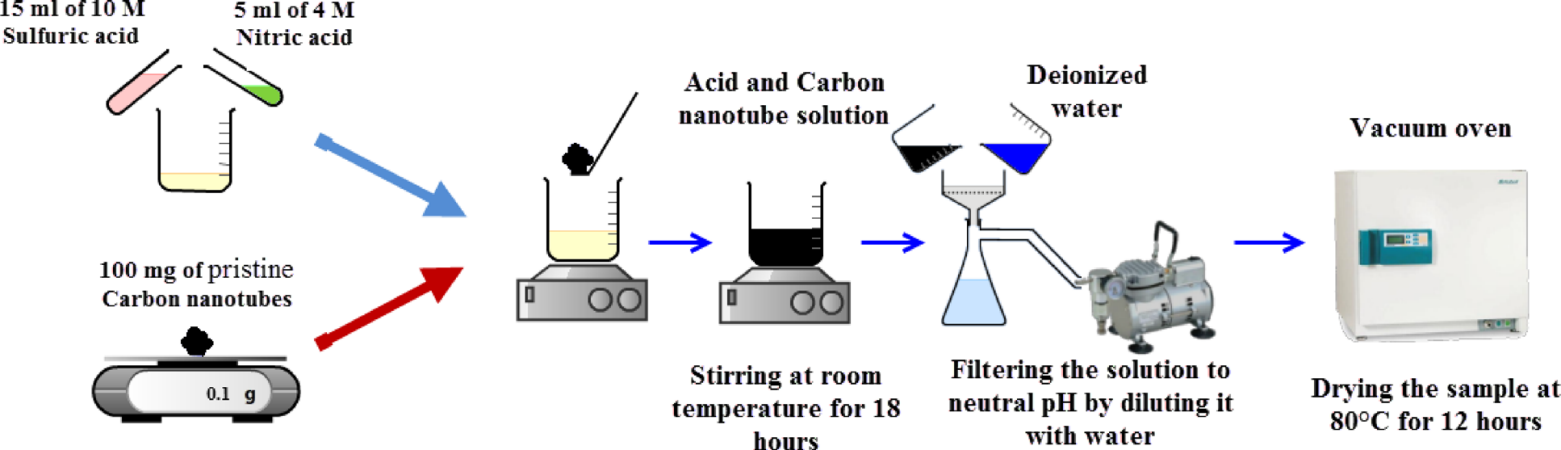
Functionalization process of the MWCNTs.

Figure 4 shows the functionalizing process of the MWCNTs using chemical oxidation method. Chemical oxidation is done using a combination of nitric acid and sulfuric acid. In order to minimize the destruction of nanotubes, nitric acid and sulfuric acid solutions were combined at 4 M and 10 M in a ratio of 3:1, respectively, to create a solution with a final volume of 20 ml. 100 mg of pristine MWCNTs were added to this solution and the mixture is stirred with magnetic stirring for 18 h at room temperature. Then, the oxidized MWCNTs are neutralized with the help of repeated cycles of dilution with deionized water and filtering the solutions until the pH reached approximately 6 or 7, and they are separated and purified from the remaining acids. After the purification process, the oxidized samples are dried at 80 °C for 12 h. Functionalization process of the MWCNTs requires 7 days in total.

#### Synthesis of Janus nanotube-silica nanostructure

**Fig. 5:**
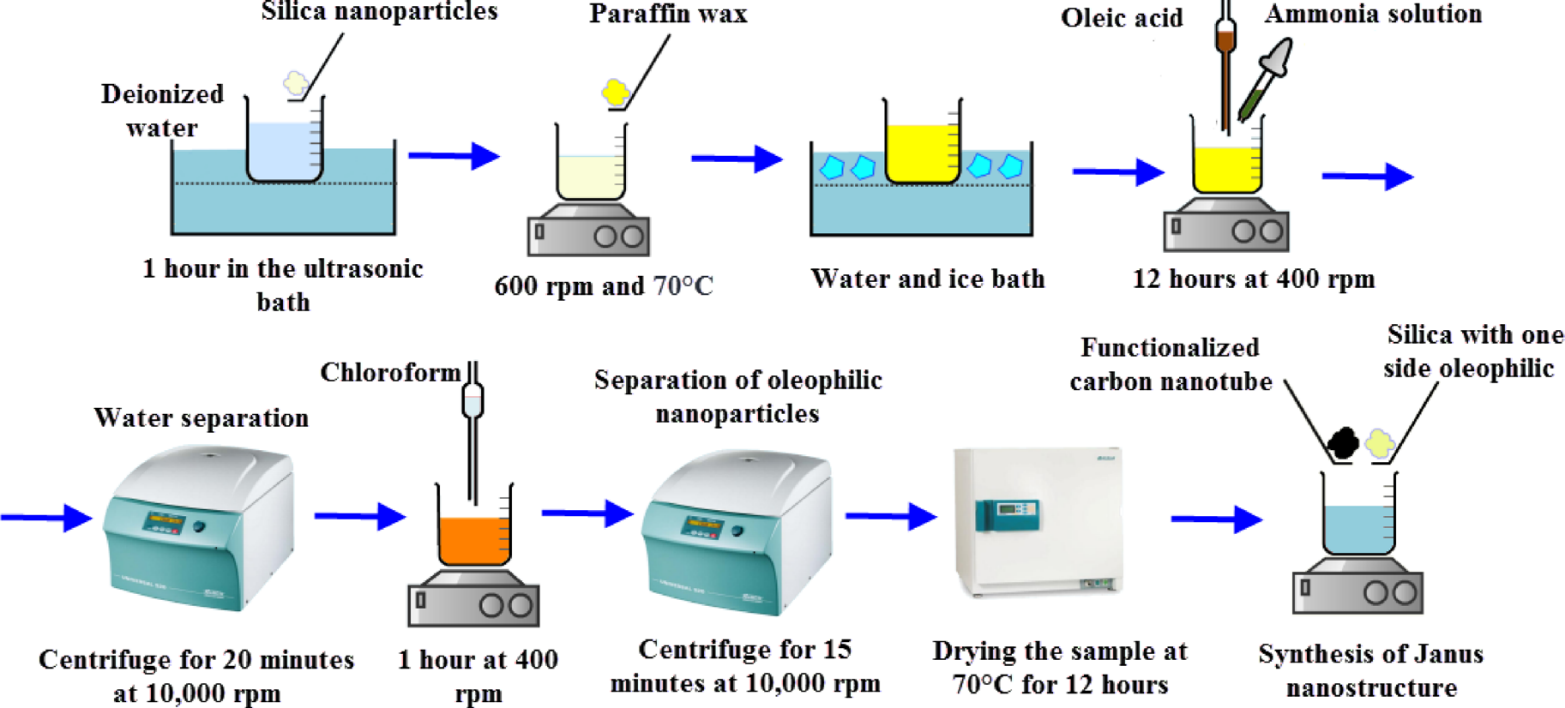
Synthesis process of nanotubes-silica Janus nanostructure.

In this study, a Janus nanostructure was synthesized by combining masking and mixing methods. Figure 5 generally shows the stages of producing Janus nanostructures by coating and mixing methods. First, with the help of coating methods and by paraffin wax, the silica nanoparticle becomes oil-wet. For this purpose, 100,000 ppm of silica nanoparticle is dissolved in 100 cc of distilled water and placed in an ultrasonic bath for 1 h. The container containing the desired sample is placed on a magnetic stirrer with a temperature near the melting temperature of paraffin wax and with a stirring speed of 600 rpm. Then, paraffin wax is slowly added to the container containing the silica nanoparticle sample in the amount of 10 times the weight of the silica nanoparticle sample. The way to add paraffin wax and its duration should be such that it becomes completely homogeneous with the environment, which lasts about 1 to 2 h. After adding all the paraffin wax, the sample is stirred with the same stirring speed as the previous one for 1.5 h so that the silica nanoparticle is covered by the paraffin wax. Next, the container containing silica nanoparticle and paraffin wax is transferred to a water and ice bath with a temperature of 0 degrees Celsius so that the paraffin particles solidify again and the silica nanoparticle are covered partially by the paraffin wax particles. The solidified paraffin wax is then washed several times with deionized water to separate the silica nanoparticle that are not covered by the paraffin. Next, 100 cc of the solution of silica nanoparticle covered by paraffin wax dissolved in water is placed on a magnetic stirrer at 400 rpm and then with the help of 5 cc of ammonia solution, the pH of the solution is brought to 12. Then, 1 cc of oleic acid is added drop by drop to the solution and the solution is allowed to stir for 12 h to make oil-wet the parts of silica nanoparticle that are not covered by paraffin wax. Next, the obtained sample is placed in a centrifuge at 10,000 rpm for 20 min and the excess water of the sample is removed. Then, in order to separate the paraffin wax attached to the nanoparticle, the paraffin wax is dissolved by adding chloroform drop by drop to the solution. Next, the solution is again poured into falcon tubes and centrifuged for 15 min at a speed of 10,000 rpm, and the precipitated part is placed in an oven at a temperature of 70 degrees Celsius to evaporate the remaining chloroform and obtain a silica nanoparticle with one side of it being oil-wet.

**Fig. 6:**
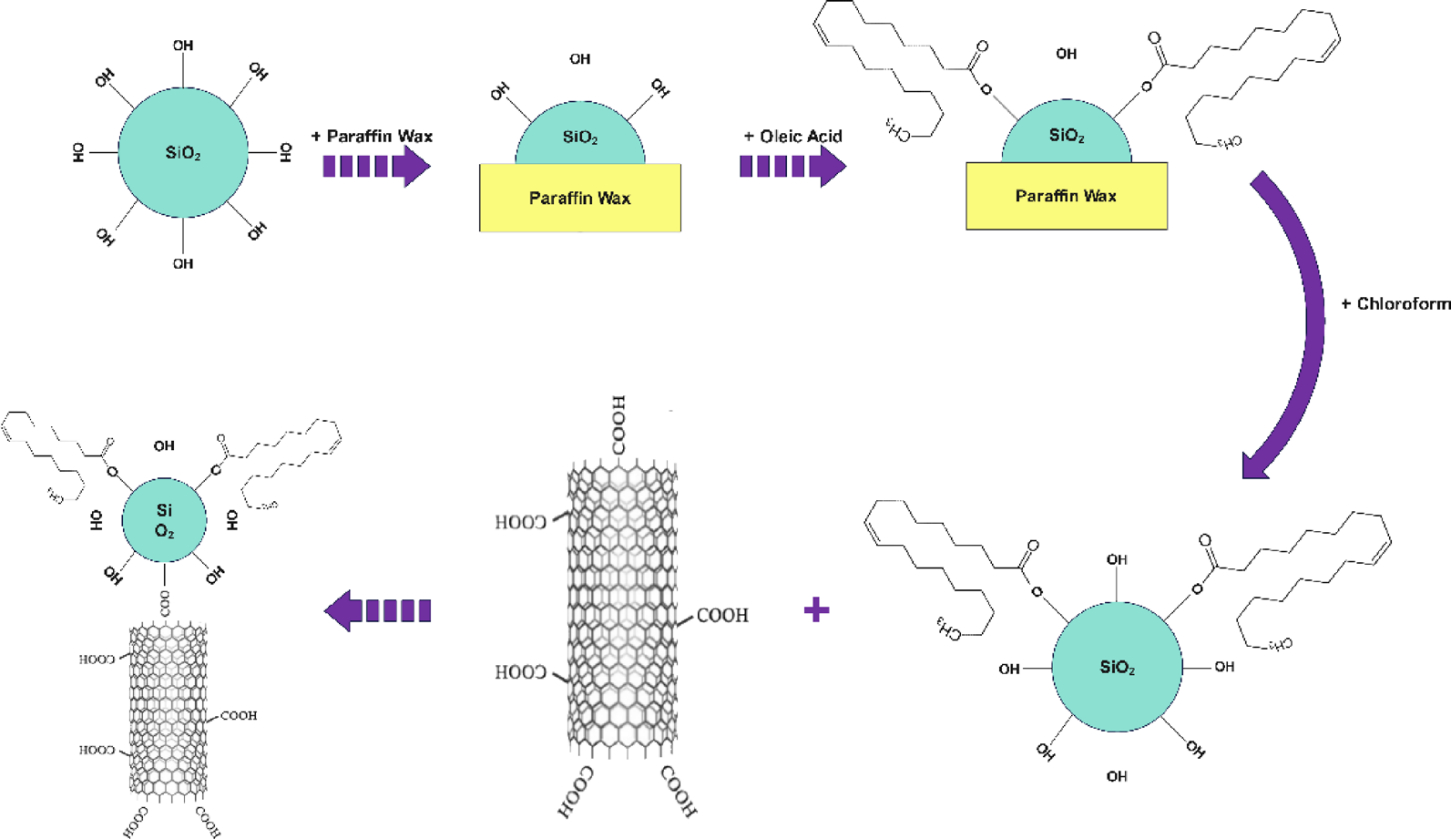
step-by-step chemical reaction representation of the synthesized nanotubes-silica Janus nanostructure.

In the following, the Janus nanostructure of nanotubes-silica is formed by the mixing method. For this purpose, depending on the ratio of silica to nanotubes, certain amounts of hydrophobic silica nanoparticle and hydrophilic nanotubes were weighed, then the hydrophilic nanotubes were gradually added to 100 cc of water on a stirrer at 500 rpm. Next, hydrophilic nanosilica along is added little by little to the desired solution and the obtained mixture is stirred for 12 h. It should be noted that the amounts of nanotubes and silica are added to the water in the ratio that is needed. In the next section, the nanostructure with different ratios of nanotubes to silica has been produced and investigated. Next, after mixing nanotubes and silica with each other in water, the obtained solution is placed in an oven with a temperature of 70 degrees Celsius to evaporate the water and obtain a Janus nanostructure (Fig. 5). A step-by-step chemical reaction representation of the synthesized Janus nanostructure is shown in Fig. 6.

#### Characterization

After the synthesis of Janus nanostructure, various methods are used to characterize it. Zeta potential test was use to quantitatively check the stability of nanotubes. FTIR, XRD, TGA and EDX analyses are used to investigate the formation of hybrid synthesized nanoparticle including MWCNT and silica. Also, FTIR analysis was used to identify the functional groups and confirm the hydrophilicity and oleophilicity of the synthesized nanostructure. TEM images are captured to see the shape and morphology of the synthesized Janus nanostructures. The average size of the synthesized crystal was estimated using XRD results. Also, with the help of TEM image analysis, the average particle size is estimated.

#### The optimal ratio of hydrophobicity and hydrophilicity of the Janus nanostructure

**Fig. 7:**
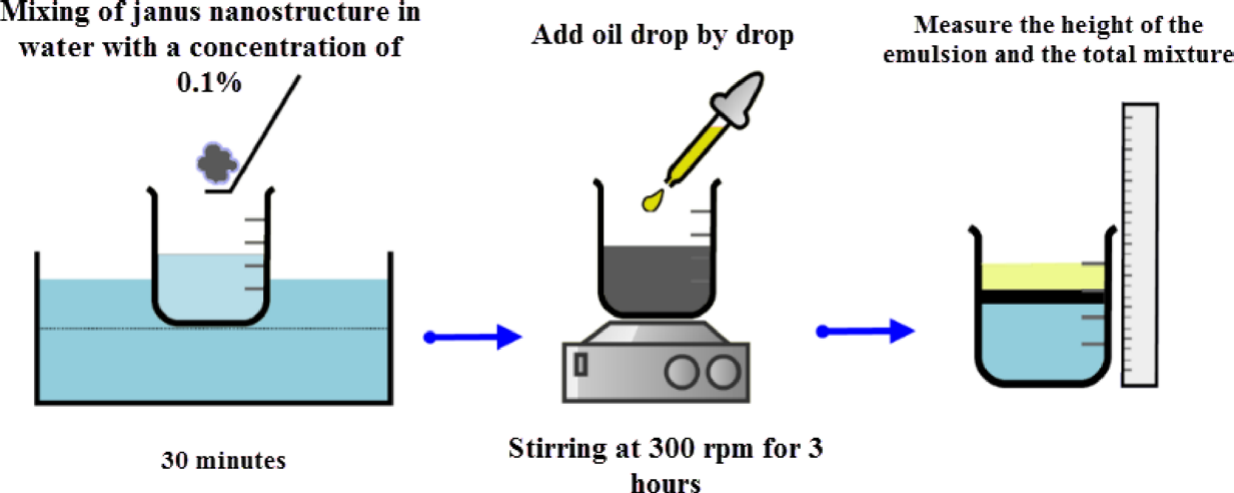
Measurement of emulsion formation in different ratios of nanotubes to silica.

The weight ratio of nanotubes to silica can play an essential role in the characteristics of Janus nanostructure. In order to find the optimal ratio of nanotubes to silica, 5 different weight ratios of nanotubes to silica of 10, 20, 30, 40 and 50% were investigated. The concentration of Janus nanostructure in all ratios was considered 0.1% by weight in water. The ratio of water to oil is 10. The relative stability of oil-in-water emulsions is evaluated by determining the percentage of gravitational separation of phases based on McClements’ method^35^. For this purpose, first, the mixture of water and Janus nanostructure is placed in an ultrasonic bath for 30 min, and homogenized. Then, the mixture of Janus nanostructure and water is placed on a magnetic stirrer at 500 rpm for 3 h, and the possibility of emulsion formation is checked by adding oil at intervals of 30 min (Fig. 7).

Phase separation is then allowed to occur through gravity and buoyancy. After a certain period, the height of each distinct boundary formed between different layers was measured with a ruler. Then the amount of emulsion stability can be expressed by the emulsification index (so-called creaming index (CI)) (Eq. 1), which is calculated as follows:

1 $$\:CI=\frac{{H}_{E}}{{H}_{t}}$$

Where $\:{H}_{E}$ is the total height of the emulsion layer and $\:{H}_{t}$ is the total height of the mixture in the container. The increase in $\:CI$ provides indirect information about emulsion stability. Also, in order to more accurately find the weight ratio of nanotubes to silica, the size of emulsions in the presence of oil and water was investigated by the naked eye and optical microscope. Like the previous case, 5 different weight ratios of nanotubes to silica of 10, 20, 30, 40 and 50% were considered and the concentration of Janus nanostructure in all ratios is 0.1% by weight in water. Also, the ratio of water to oil is 10 as before. Then, a photo is taken from the formed emulsion samples by optical microscope. The size of the emulsion indicates the reduction of surface tension and stability of emulsions and will help in finding the best ratio of nanotubes to silica.

## Results and discussion

### Results of the functionalization of MWCNT

**Fig. 8:**
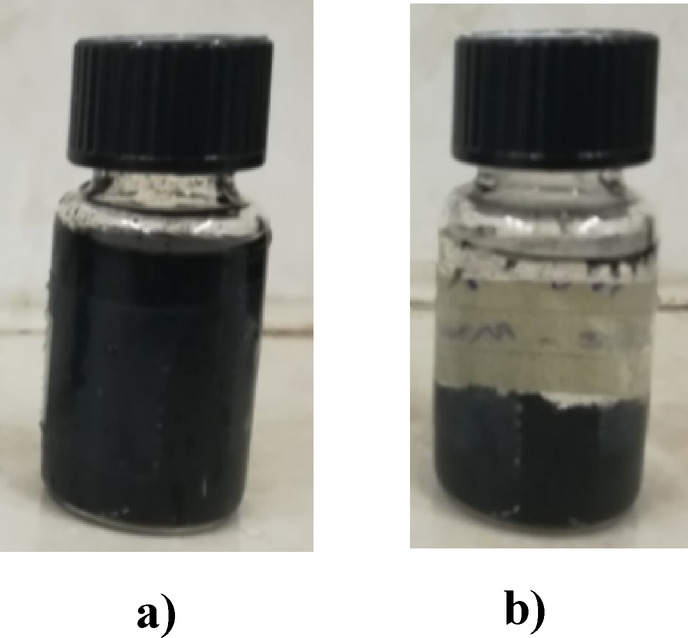
Hydrophilicity of pristine MWCNT sample after (**a**) zero residence time, (**b**) 1 min residence time.

Nanotubes are hydrophobic due to their carbon nature and the presence of van der Waals attraction forces between the tubes, so they show little solubility in water, and even the use of ultrasound waves does not improve their solubility. Figure 8 shows the solubility of 20 mg of pristine MWCNT in 10 mL of deionized water after 15 min in an ultrasonic bath. After 1 min, the sample starts to deposit on the bottom of the container and it clearly shows that the nanotube is not hydrophilic in the normal state.

**Fig. 9:**
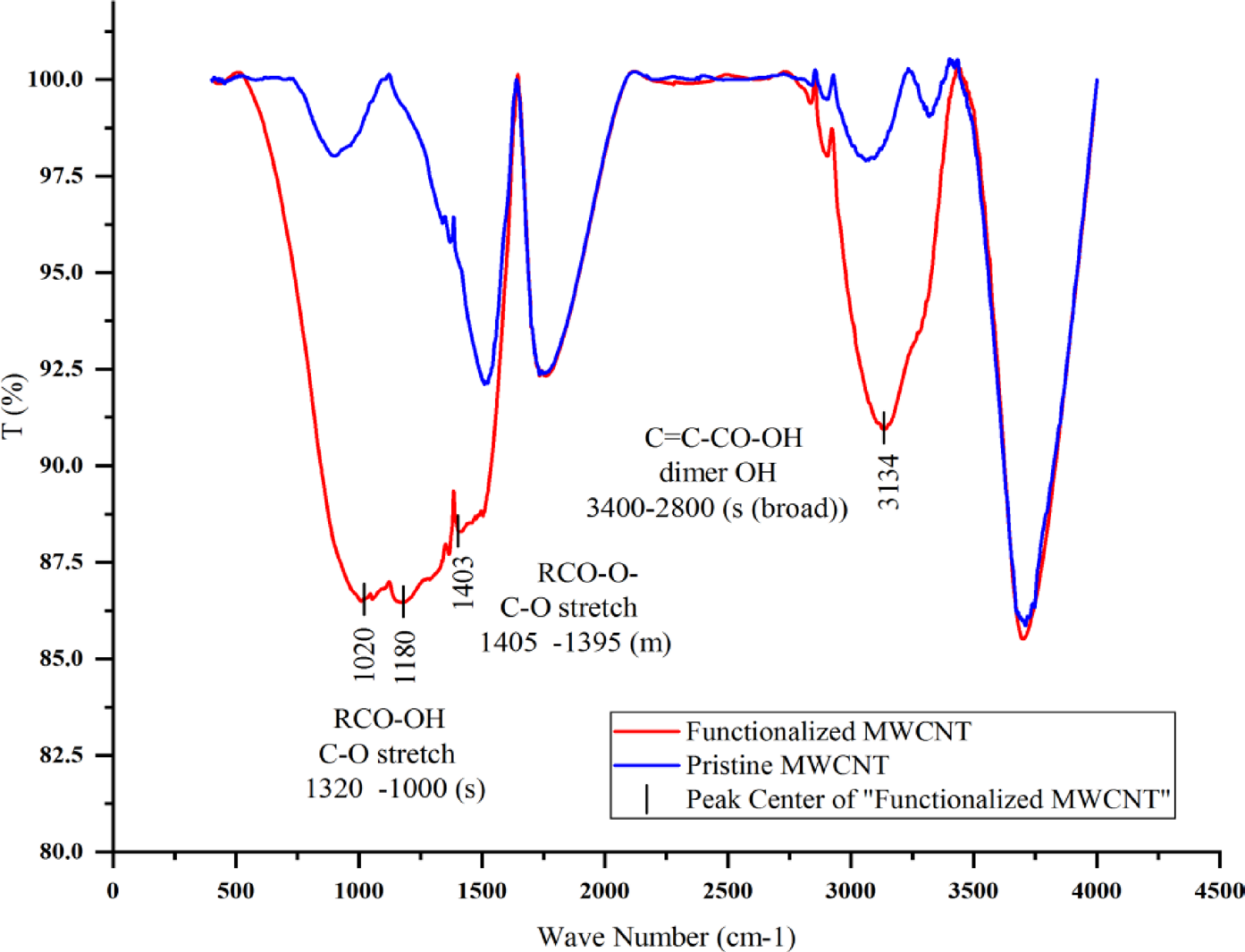
FTIR analysis of pristine MWCNT (blue color) and functionalized MWCNT (red color).

However, nanotubes functionalized by chemical methods show hydrophilic behavior, and the results of chemical functionalization of nanotubes will be discussed in the following. The acid washing process leads to the formation of O-H groups on the surface of the nanotubes, which leads to the creation of hydrogen bonds with water molecules. Figure 9 shows the FTIR analysis of the samples of pristine multi-walled nanotubes and multi walled nanotubes chemically functionalized by sulfuric acid and nitric acid with a ratio of 3:1. A PerkinElmer Frontier IR-FIR FTIR spectrometer was used to perform precise infrared spectroscopy and rapid data analysis. TGA tests were performed by TA Q600 instrument.

High symmetry in non-functionalized nanotubes produces very weak infrared signals, which is due to the weak charge state difference between carbon atoms. The peaks between 2800 and 3500 cm^− 1^ are characteristic peaks of the O-H dimer bonds of carboxylic acid groups^36^. The peak at 1403 cm^− 1^ is related to C-O stretching and represents carboxylic groups, which are created due to surface oxidation. The peaks in the range between 1000 and 1320 cm^− 1^ are characteristic peaks related to C-O stretching bonds of carboxylic acid group^37^. The FTIR results confirm the formation of C-O, O-H and -COOH functional groups on the surface of multi-walled nanotubes, which leads to the addition of the ability to create hydrogen bonds with water molecules and create hydrophilic properties in MWCNT.

The distribution of functional groups on the surface of MWCNTs is not uniform. Acid treatments can introduce a high density of carboxyl groups but may also cause defects and asymmetry in the structure, leading to an uneven distribution across the nanotube surface^38^. Research indicates that the density of functional groups on MWCNTs is affected by both the strength and concentration of the acid employed during the functionalization process. This variability results in an uneven distribution, with certain areas exhibiting a greater concentration of functional groups than others^39^. Studies employing electron energy-loss spectroscopy (EELS) have revealed that functional groups, especially oxygenated species such as carboxyl groups, are distributed across individual MWCNTs at the nanometer scale. This distribution suggests that certain regions of the nanotube surface may have elevated concentrations of specific functional groups, while other areas may show a lower density^40^.

**Fig. 10:**
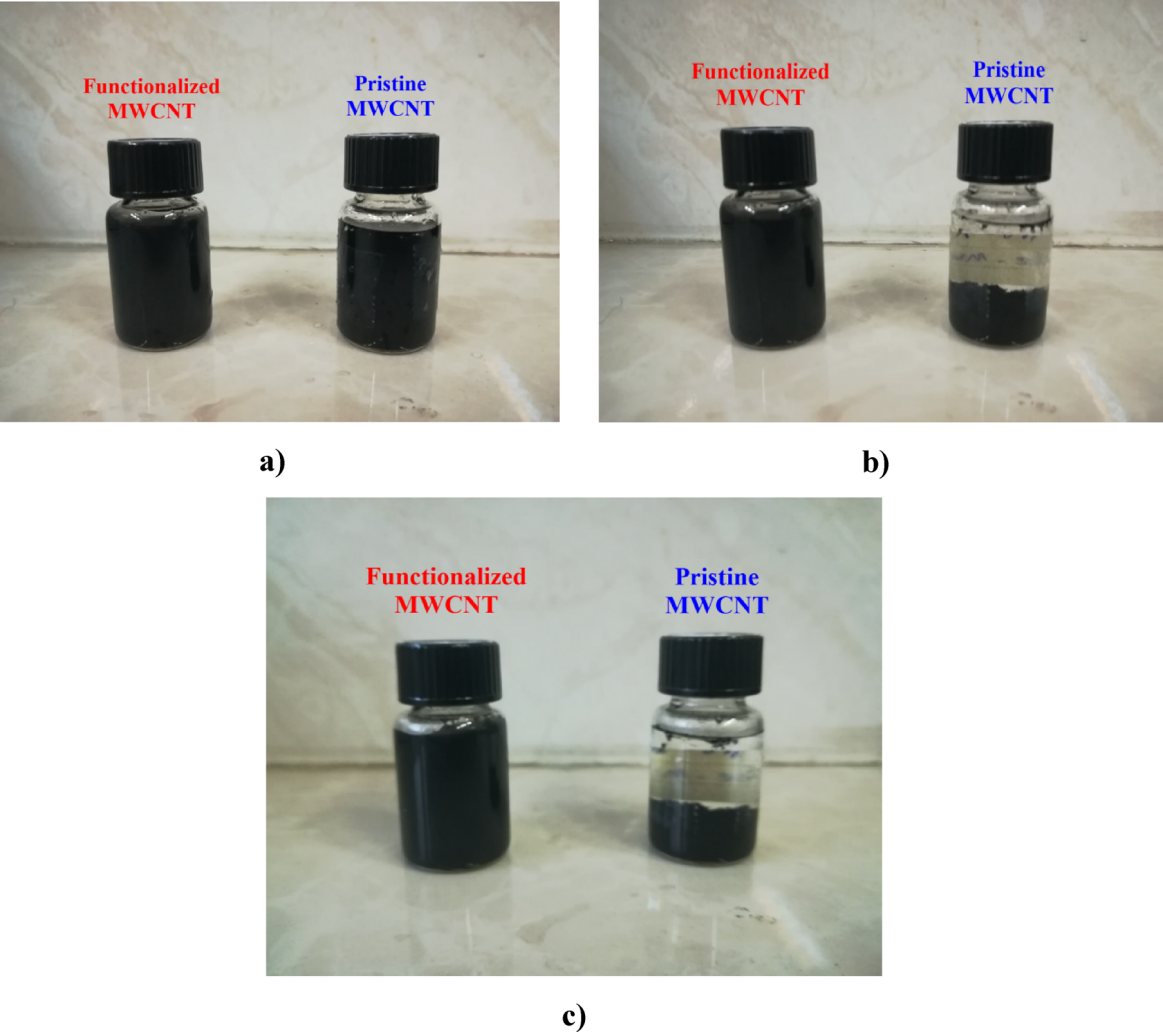
Hydrophilicity of pristine and functionalized MWCNT, (**a**) zero residence time, (**b**) 1 min residence time, (**c**) 6 days residence time.

To compare the hydrophilicity of nanotubes before and after functionalization; 20 mg of pristine nanotube and functionalized nanotube are poured into 10 ml of water in two separate containers and placed in an ultrasonic bath for 15 min. Then the samples are given time to check the stability of the samples. As shown in Fig. 10, the pristine nanotube sample begins to precipitate after 1 min, but the functionalized nanotube sample is still stable in water after 6 days, which indicates that the functionalized nanotube is well hydrophilic.

**Fig. 11:**
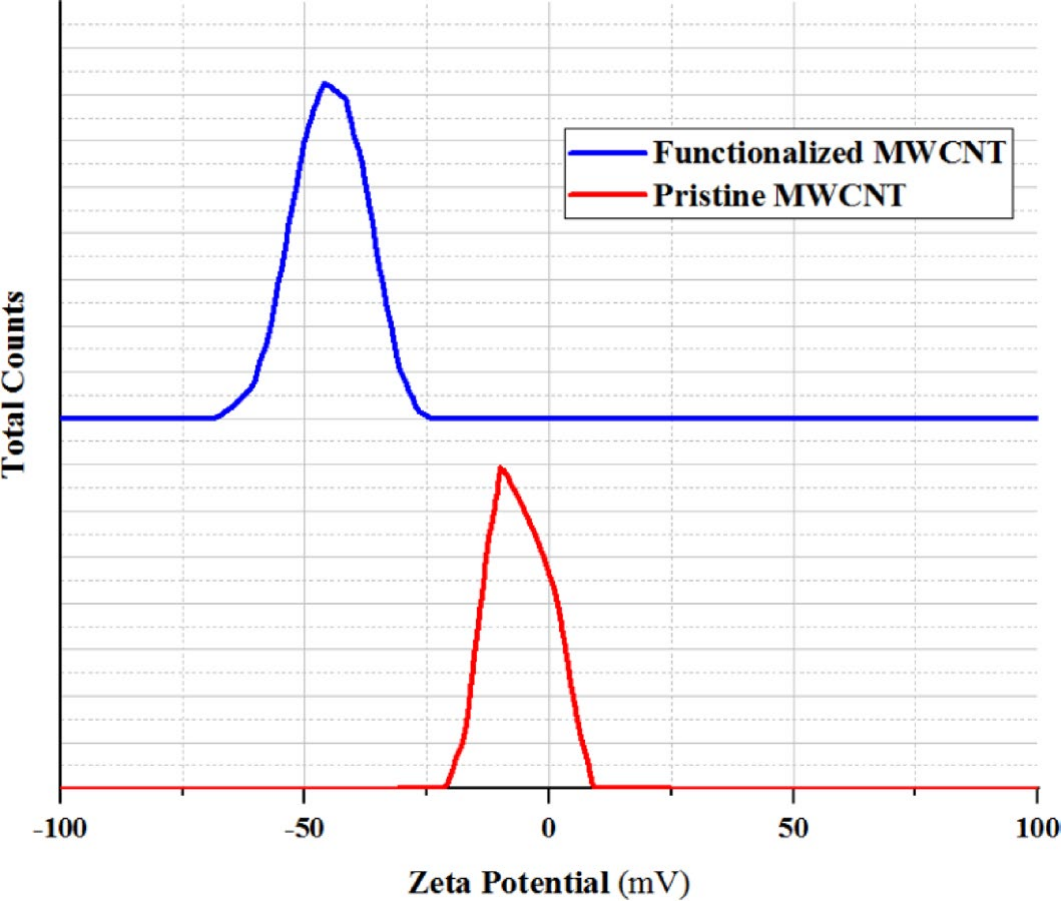
Zeta potential of pristine MWCNT (red) and functionalized MWCNT (blue).

In order to quantitatively check the stability of nanotubes, zeta potential test was performed on pristine MWCNT and functionalized MWCNT samples by Horiba SZ100 analyzer, the results of which are shown in Fig. 11. In this test, if the value of the zeta potential is less than a certain value, the particles will stick together due to the increase of van der Waals attraction forces and cause instability of the solution^41^. Zeta potential with absolute values of 0–10, 10–20, 20–30 and more than 30 mV indicates unstable, relatively stable, moderately stable and very stable solution, respectively^42^. As can be seen from the results of the Fig. 11, the absolute value of the zeta potential value for the pristine MWCNT is 9.9 mV, which indicates the instability of the solution. But after the functionalization of pristine MWCNT, the absolute value of zeta potential has increased up to 44.6 mV and the solution has become stable.

### Results of the functionalization of silica

**Fig. 12:**
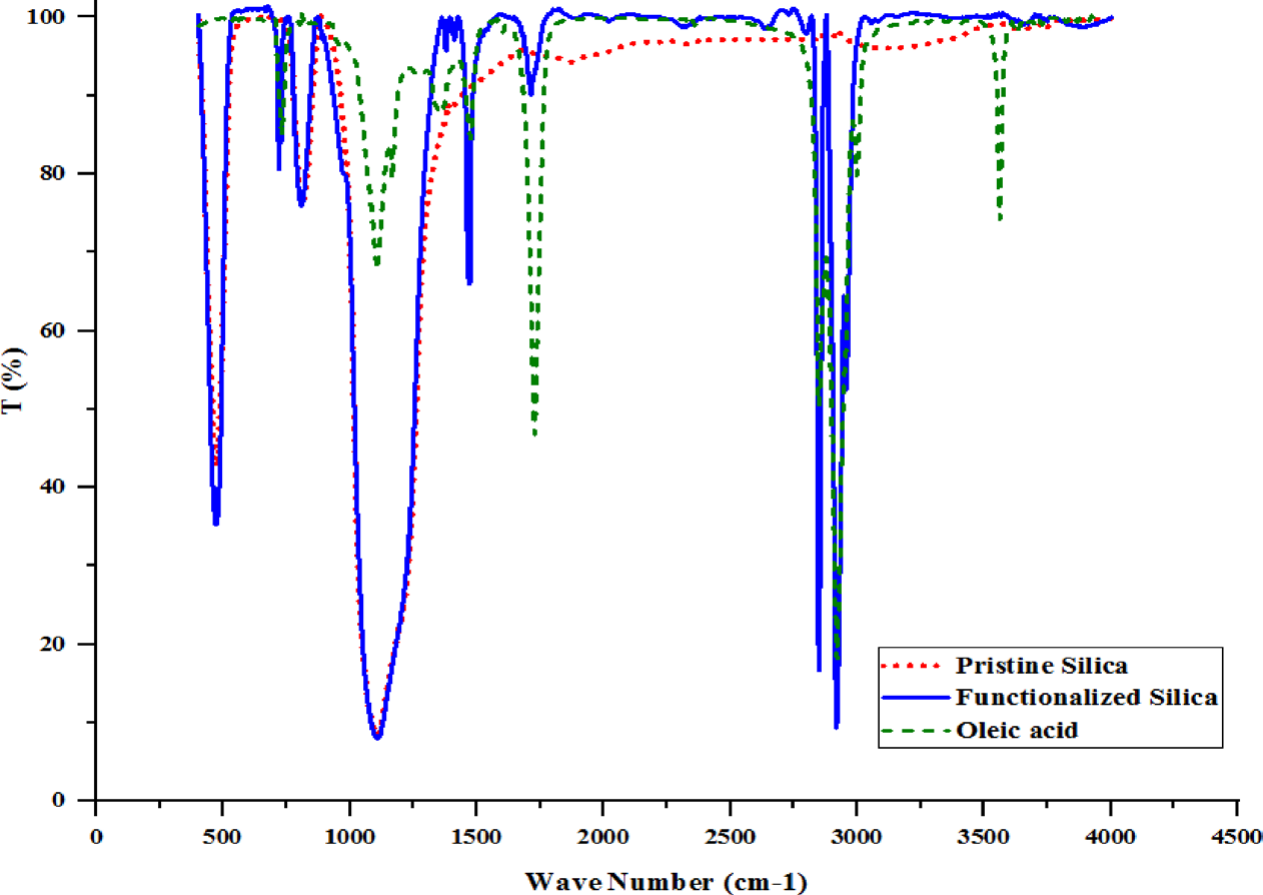
FTIR analysis of pristine silica (red color), functionalized silica (blue color) and oleic acid (green color).

In order to better understand the surface modification of silica nanoparticle and make it oleophilic with the help of oleic acid, the infrared spectrum results of silica nanoparticle, functionalized silica nanoparticle and oleic acid are shown in Fig. 12. The absorption spectrum at 469 cm^− 1^ and 807 cm^− 1^ is related to Si-O and Si-OH connections in silica nanoparticle, respectively. Also, the absorption peak at 1107 cm^− 1^ corresponds to Si-O-Si in silica nanoparticle^43^. Also, the absorption spectrum at 3117 cm^− 1^ is related to OH in silica nanoparticle, which usually occurs in the range of 2800 cm^− 1^ to 3400 cm^− 1^^44^. The peak observed at 3562 cm^− 1^ on oleic acid is attributed to the O-H stretching vibrations associated with carboxylic acid groups (-COOH)^45^. The peak observed at 3000 cm^− 1^ on oleic acid, which exhibits a moderate sharpness and overlaps with the O-H stretching band, is ascribed to the C-H stretching associated with the vinyl group^45^. The presence of two distinct peaks at 2851 cm^− 1^ and 2920 cm^− 1^ on oleic acid corresponds to the symmetric and asymmetric stretching vibrations of -CH_2_ groups, respectively^46^. The prominent peak observed at 1728 cm^− 1^ on oleic acid is primarily associated with the C = O stretching, while the peak at 1473 cm^− 1^ is linked to the -CH_2_- of the alkanes^45^. Absorption at 720 cm^− 1^ is related to the RCH_2_CH_3_ bond of alkanes and RCH = CHR alkanes in oleic acid, which are usually in the range of 720 cm^− 1^ to 725 cm^− 1^ and 675 cm^− 1^ to 725 cm^− 1^ respectively.

In the functionalized silica nanoparticle, the absorption spectrum at 471 cm^− 1^, 807 cm^− 1^ and 1104 cm^− 1^ is respectively related to Si-O, Si-OH and Si-O-Si connections in the silica nanoparticle.

The absorbance at 720 cm^− 1^ is related to the RCH_2_CH_3_ bond of alkanes and RCH = CHR alkanes in oleic acid. Also, the absorption spectrum at 1473 cm^− 1^ is related to the -CH_2_- bonds of alkanes, which does not have such a peak in pristine silica nanoparticle. The presence of oleic acid functionalized silica nanoparticle was characterized by a diminished peak at 1715 cm^− 1^, which serves as evidence for the ester linkage formed between the silanol group and the carboxylic group of oleic acid. Consequently, it can be inferred that the –COOH group of oleic acid has interacted with the surface –OH groups of nanosilica, resulting in the formation of a carboxylate bond^47^.

Furthermore, the absorption peaks identified at 2849 cm^− 1^ and 2919 cm^− 1^ are indicative of the stretching vibrations associated with the -CH_2_ group^46^, which are absent in the pristine silica nanoparticle. This observation suggests the presence of long alkyl chains in the functionalized silica nanoparticle treated with oleic acid. Consequently, the FTIR data confirm that the silica nanoparticle has been effectively functionalized with oleic acid.

### Characterization of Janus nanostructure

#### FTIR test results

**Fig. 13:**
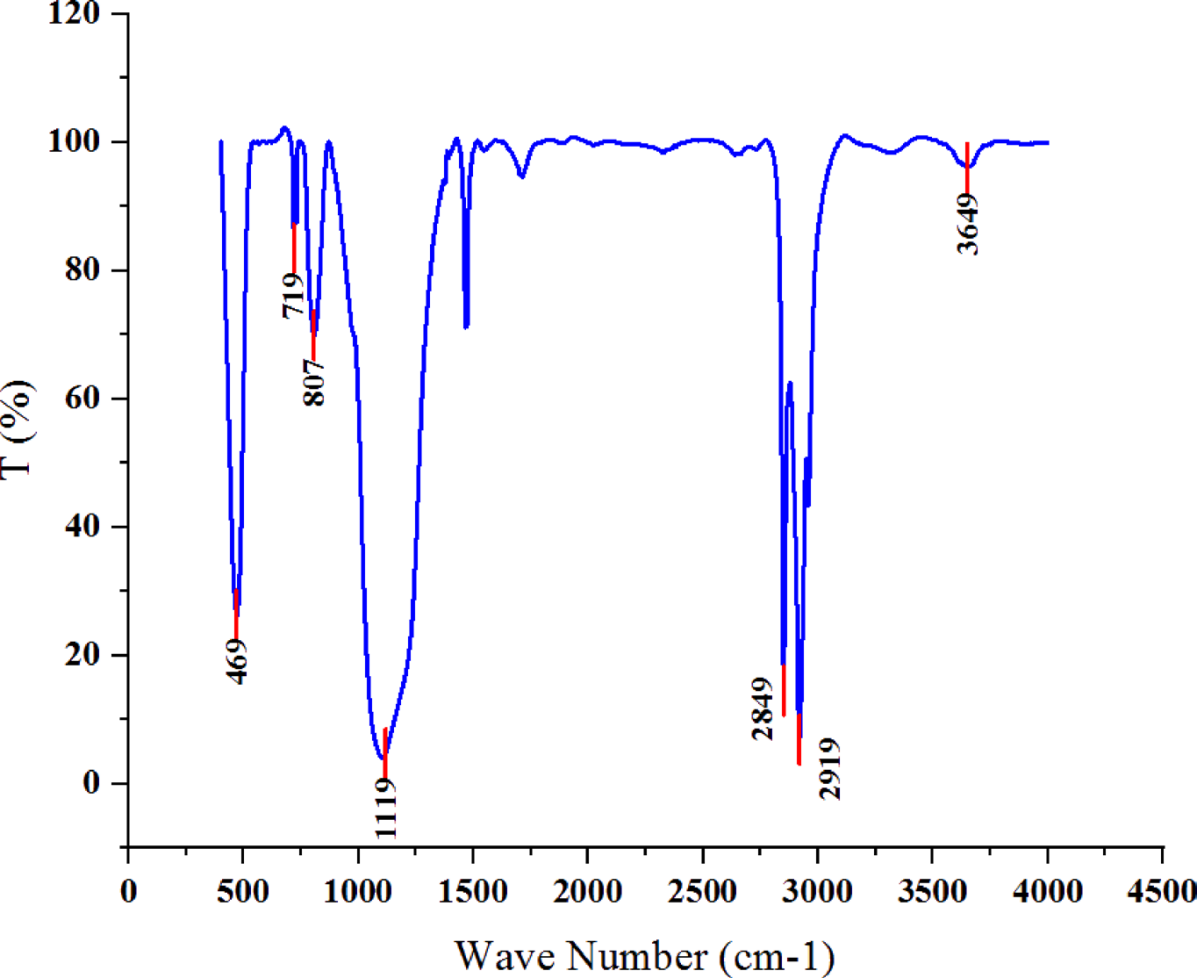
FTIR analysis of nanotube-silica Janus nanostructure.

FTIR test was performed on the synthesized Janus nanostructure. Figure 13 shows the results of the infrared spectrum of the synthesized nanostructure. The index peak in the range of 3649 cm^− 1^ is probably related to the nanotube and the presence of carboxylic group in the primary structure of the functionalized nanotube considering that it is also located on the functionalized nanotube (Fig. 9). The absorption spectrum at 469 cm^− 1^ and 807 cm^− 1^ is related to Si-O and Si-OH bonds in silica nanoparticle, respectively. Also, the absorption peak at 1119 cm^− 1^ is related to Si-O-Si in silica nanoparticle. Also, absorption spectrum at 719 cm^− 1^ is related to RCH2CH3 alkanes and RCH = CHR alkanes bond in oleic acid, which are usually in the range of 720 cm^− 1^ to 725 cm^− 1^ and 675 cm^− 1^ to 725 cm^− 1^ respectively. The 2849 cm^− 1^ peak may be related to the CH_2_ bond of alkane oleic acid or to the OH dimer bond of the carboxylic acid group of the functionalized nanotube. In the same way, the 2919 cm^− 1^ peak can be related to the CH stretch bond, RCH_2_CH_3_ of alkane oleic acid or to the OH dimer bond of the carboxylic acid group (RCOOH or C = C-CO-OH) of the functionalized nanotube.

#### XRD test results

**Fig. 14:**
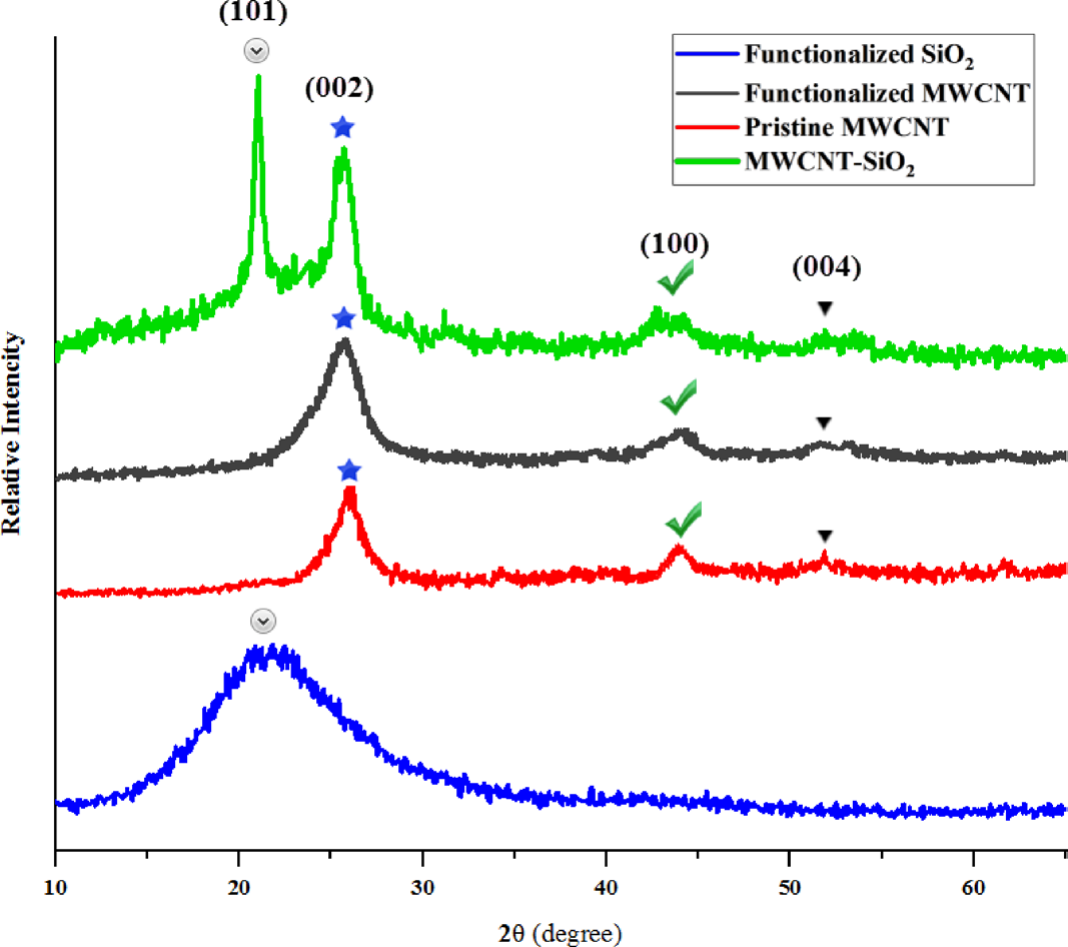
The results of XRD analysis of the Janus nanostructure sample, pristine MWCNT, functionalized MWCNT and functionalized silica.

Figure 14 shows the XRD pattern of the synthesized Janus nanostructure, pristine MWCNT, functionalized MWCNT and functionalized silica. XRD tests were performed using Philips PW 1730/10 device. Although the XRD patterns of pristine MWCNT and functionalized MWCNT share a similar shape, the diffraction lines for functionalized MWCNT are noticeably broader. The peaks identified at 26° for pristine MWCNT and at 25.7° for functionalized MWCNT are indicative of a graphite-like structure present in the MWCNT. The notable peak detected at 26° is associated with the hexagonal graphite C (002), as can be recognized^48^. Furthermore, additional specific peaks of crystalline graphite appear at approximately 43° and 53°, corresponding to the XRD peaks of C (100) and C (004) graphite, respectively. Any reduction in crystallinity order in MWCNT by oxidation would result in broader XRD peaks in functionalized MWCNT^49^. A broad peak at 22° (101) confirmed the amorphous nature of the functionalized silica^50^. Since oleic acid does not have a crystalline state, it does not change the XRD pattern of the functionalized silica relative to the XRD pattern of the silica. It just may absorb some X-rays and reduce the height of the peaks. Therefore, in order to not crowd Fig. 14, XRD pattern of pristine silica was omitted. In the XRD results of the synthesized sample, 3 peaks of graphite (indicating functionalized MWCNT) and a peak of functionalized silica (at 22° (101)) can be identified. Therefore, the matching of the peaks of the synthesized samples with the standard patterns of graphite and silica indicates the formation of hybrid synthesized nanoparticle including MWCNT and silica.

In the following, the crystal size of the synthesized samples was estimated using the XRD results and the Scherer equation. Scherer proposed the following relationship to calculate the crystal size, where $\:\lambda\:$ is the X-ray wave length, $\:{\beta\:}_{d}$ is the full width at half of the maximum height (FWHM), θ is the diffraction angle of the peak, and D is the crystal size.

2 $$\:D=\frac{0.9\:\lambda\:}{{\beta\:}_{d}\text{cos}\theta\:}$$

According to the Eq. 2, the crystal size can be obtained for each peak and the average crystal size of different peaks can be calculated as the crystal size of the sample. Table 3 shows the size of different peaks. According to the results of the table, the average size of the synthesized crystal is estimated to be about 13 nm for the Janus nanostructure sample of the synthesized nanotube-silica.

**Table 3:** Size Estimation of the Janus nanostructure with the help of XRD test results.

Position. [°2Theta.]	Height [cts]	FWHM [°2Theta.]	Crystallite Size [Å]
21.60	905.42	0.72	118.76
23.96	338.41	1.09	79.81
26.21	681.10	1.15	77.06
34.66	65.24	0.29	327.65
44.09	94.93	2.3	48.12

#### TEM test results

**Fig. 15:**
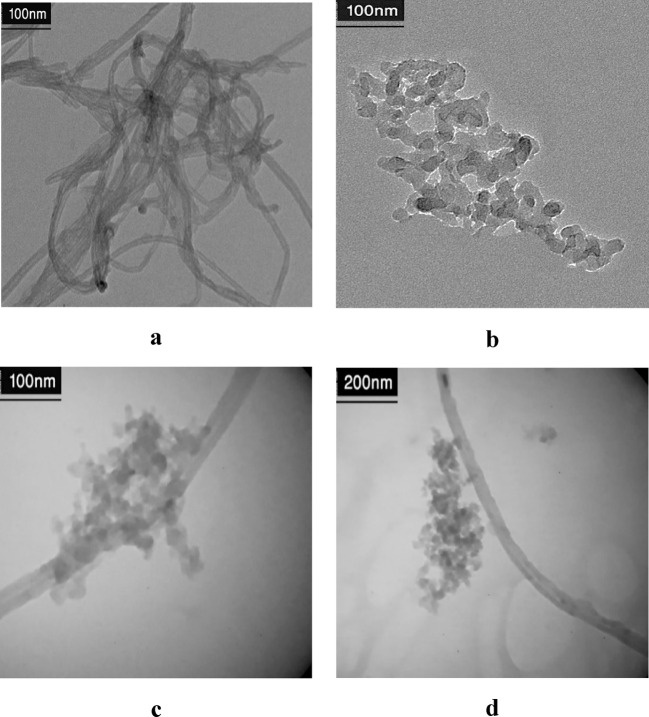
TEM images of modified nanotube (**a**), modified silica (**b**) and Janus MWCNT-silica nanostructure (**c** and** d**).

TEM analysis was performed by Philips CM-120 device. TEM analysis of modified nanotube, modified silica, and Janus MWCNT-silica nanostructure is shown in Fig. 15. It is well shown in Fig. 15c and d how the silica nanoparticle is attached on the MWCNT structure.

**Fig. 16:**
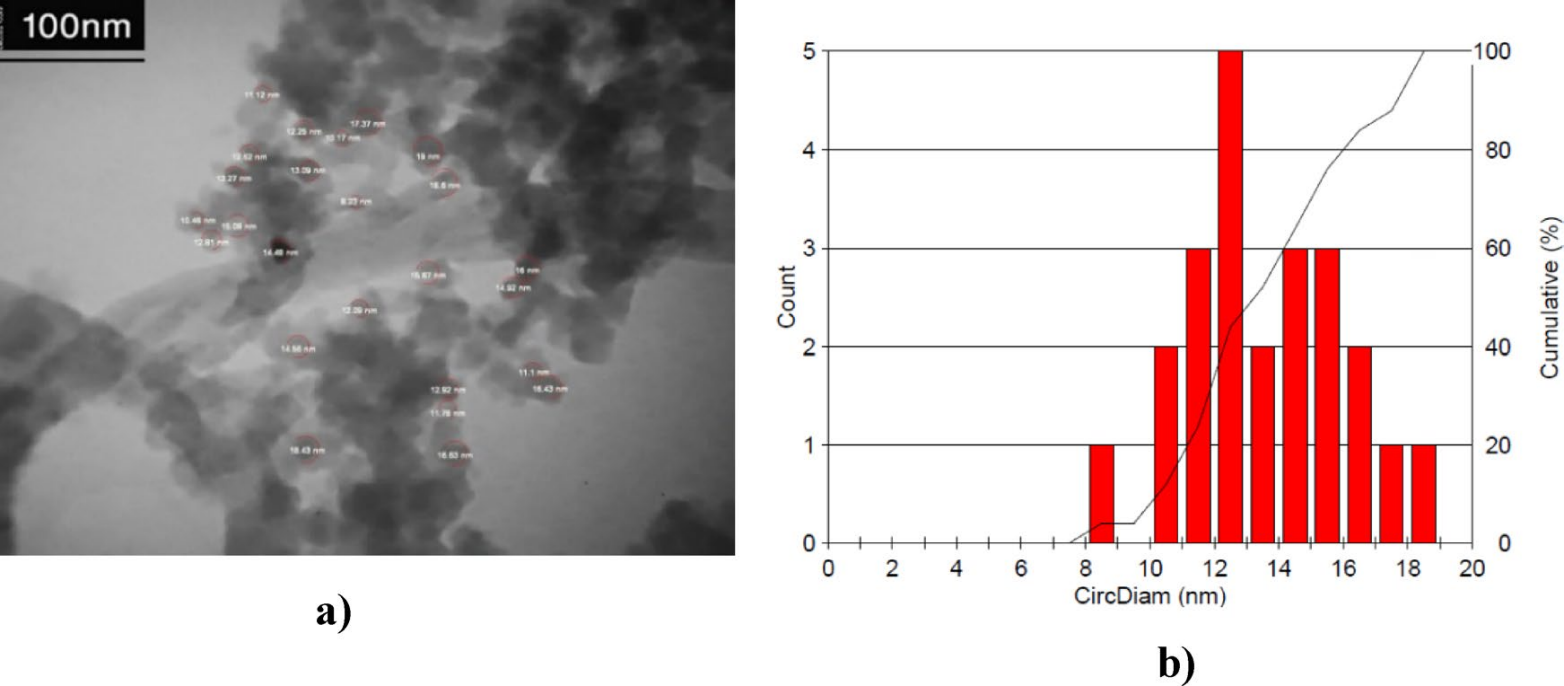
Size estimation of the Janus nanostructure with the help of TEM test results, (**a**) analysis of images, (**b**) particle size distribution.

Also, by analyzing TEM images by Image J software, the size of nanoparticle can be estimated. Figure 16a shows the analysis of the TEM images and Fig. 16b shows the particle size distribution. According to the results, the average size of nanoparticle is 14 nm. Some agglomeration of the nanoparticle can be observed. Agglomeration of nanoparticle can impact the properties and functionality of the composite. It can lead to reduced dispersion and increased particle size, which may alter the intended hydrophobic/hydrophilic balance of the Janus structure. Since, the silica nanoparticle are modified with hydrophobic agents, they can contribute to the hydrophobic nature of the Janus structure, Therefore, the agglomeration of nanoparticle in one place causes the hydrophobicity in one place to increase excessively. Employing sonication or other mechanical dispersion methods may lead to a uniform distribution of nanoparticle on MWCNTs, reducing the likelihood of agglomeration. Also, using stabilizing agents or surfactants that can interact with both the silica nanoparticle and the MWCNTs, may lead to a stable dispersion and preventing aggregation. Adjusting the pH and solvent conditions during synthesis to optimize the interaction between silica nanoparticle and MWCNTs can minimizing agglomeration by controlling electrostatic forces and surface charges.

#### TGA and EDX test results

**Fig. 17:**
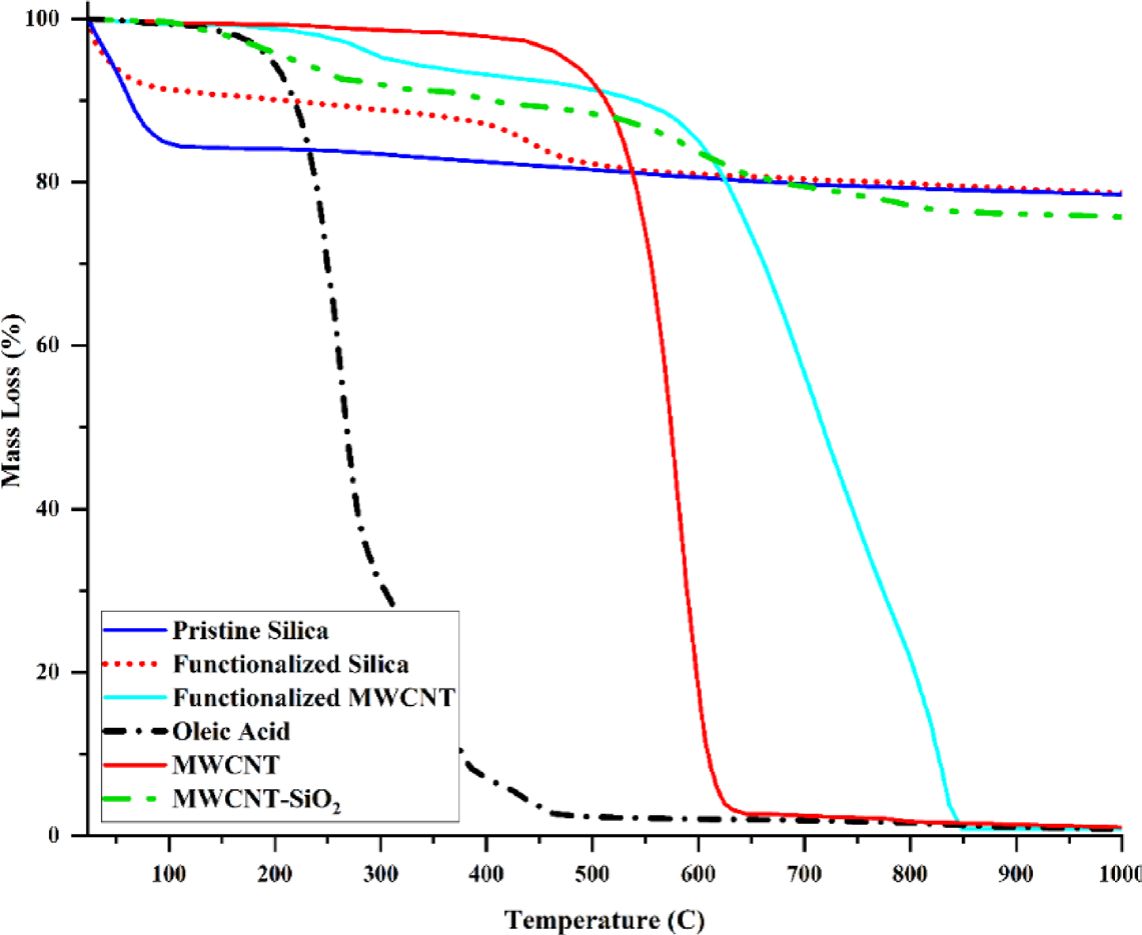
TGA of oleic acid, pristine silica, pristine MWCNT, functionalized silica, functionalized MWCNT and MWCNT-SiO_2_ Janus nanostructure.

Figure 17 shows the thermogravimetric analysis (TGA) of oleic acid, pristine silica, pristine MWCNT, functionalized silica, functionalized MWCNT and MWCNT-SiO_2_ Janus nanostructure. TGA tests were performed by TA Q600 instrument. The TGA of silica reveals two separate stages of mass loss. The initial stage, occurring at temperatures below 120 °C, is characterized by a rapid decline, likely resulting from the evaporation of physisorbed water from the silica surface^51^. The subsequent stage is more gradual and is thought to be associated with the slow condensation of silanol groups^51^. The weight loss percentage of oleic acid functionalized silica is recorded at 10% when subjected to a temperature of 116 °C. This weight reduction is attributed to the evaporation of moisture that is adsorbed on the surface hydroxyl groups of the silica. According to the TGA results, the oleic acid functionalized silica displayed reduced water absorption relative to the pure silica sample, revealing an 8% weight loss difference at a temperature of 116 °C. Oleic acid undergoes decomposition at temperatures below 500 °C. The functionalization of silica particles with oleic acid leads to the formation of ester bonds between surface hydroxyl groups and the carboxylic groups of oleic acid. This process results in a hydrophobic surface that inhibits the adsorption of water molecules onto the silica. There remain several hydroxyl groups that have not been altered by oleic acid. Consequently, water molecules are able to bind to the regions where free hydroxyl groups are present. MWCNTs exhibit a single-step decomposition, with weight loss initiating at 450 °C. In contrast, functionalized MWCNTs experience weight loss at a significantly lower temperature of approximately 200 °C. The weight loss associated with MWCNT-COOH demonstrates a two-step decomposition process. MWCNT-SiO_2_ started to decompose right from the beginning of the experimental process. Functionalized nanotube undergoes complete decomposition at temperatures exceeding 850 °C. Functionalized silica maintains its stability at temperatures exceeding 550 °C. As a result, one can assess the silica concentration in MWCNT-SiO_2_ at temperatures above 850 °C, with an estimated value of around 80 weight%.

**Fig. 18:**
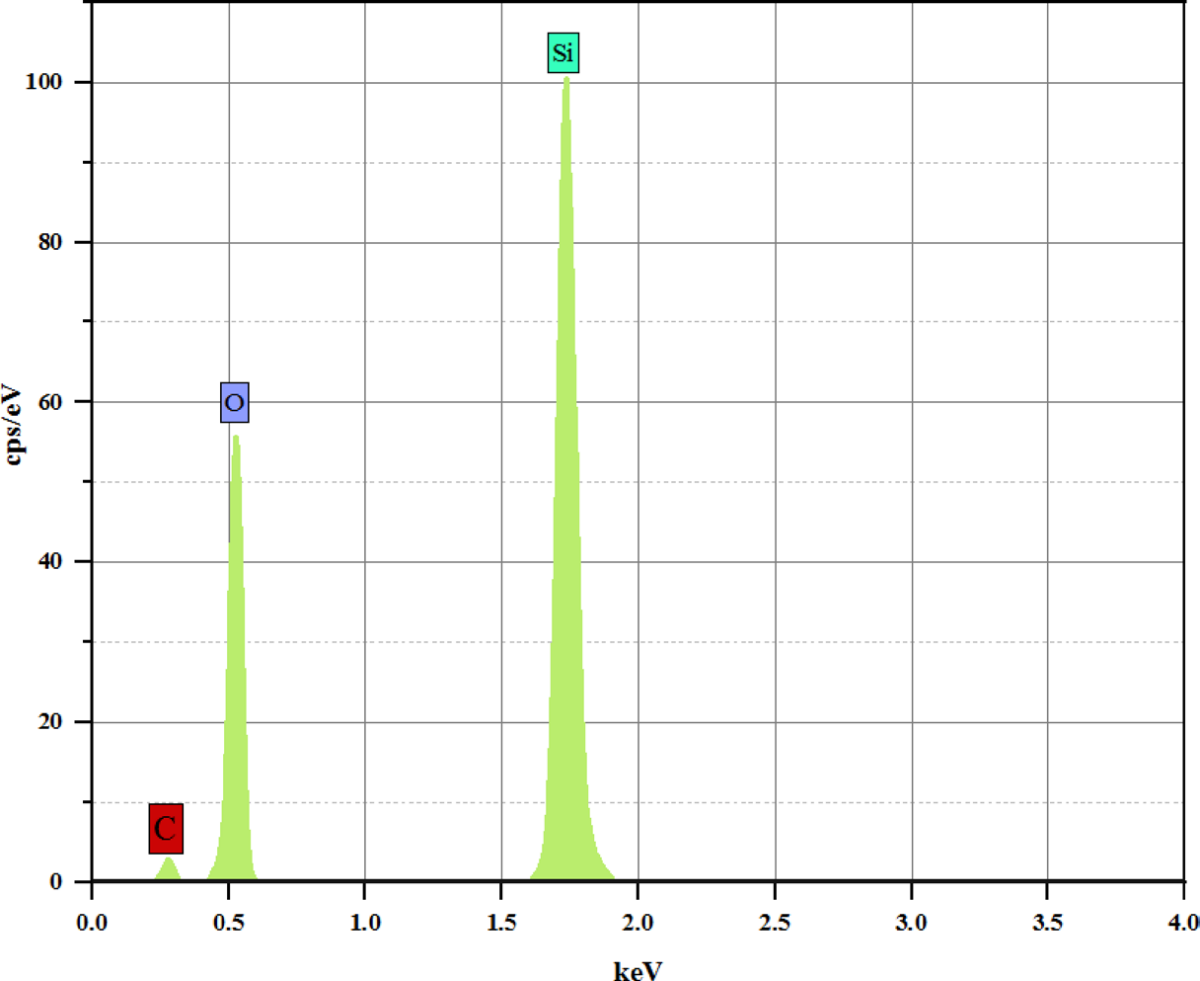
EDX spectra of MWCNT-SiO_2_ Janus nanostructure.

Also, Fig. 18 shows the EDX analysis of the MWCNT-SiO_2_ Janus nanostructure in the ratios of nanotube to silica 20%. TESCAN VEGA3 devise was used for EDX elemental analysis. The data presented in Figure indicates that the elemental composition of the MWCNT-SiO^2^ Janus nanostructure comprises 50.94 wt% oxygen, 40.81 wt% silicon, and 8.25 wt% carbon.

### Investigating the hydrophilic/hydrophobic properties of the Janus nanostructure

**Fig. 19:**
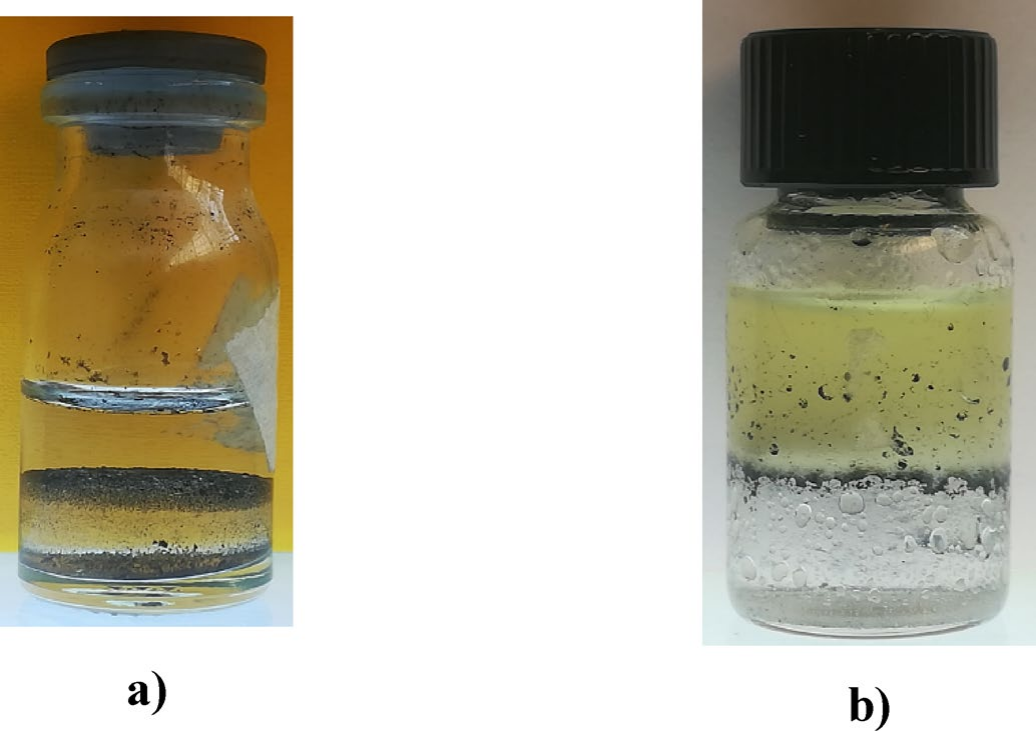
Investigation the hydrophilic/hydrophobic properties of the synthesized nanostructure (**a**) The interface between chloroform and water, (**b**) The interface between engine oil and water.

**Fig. 20:**
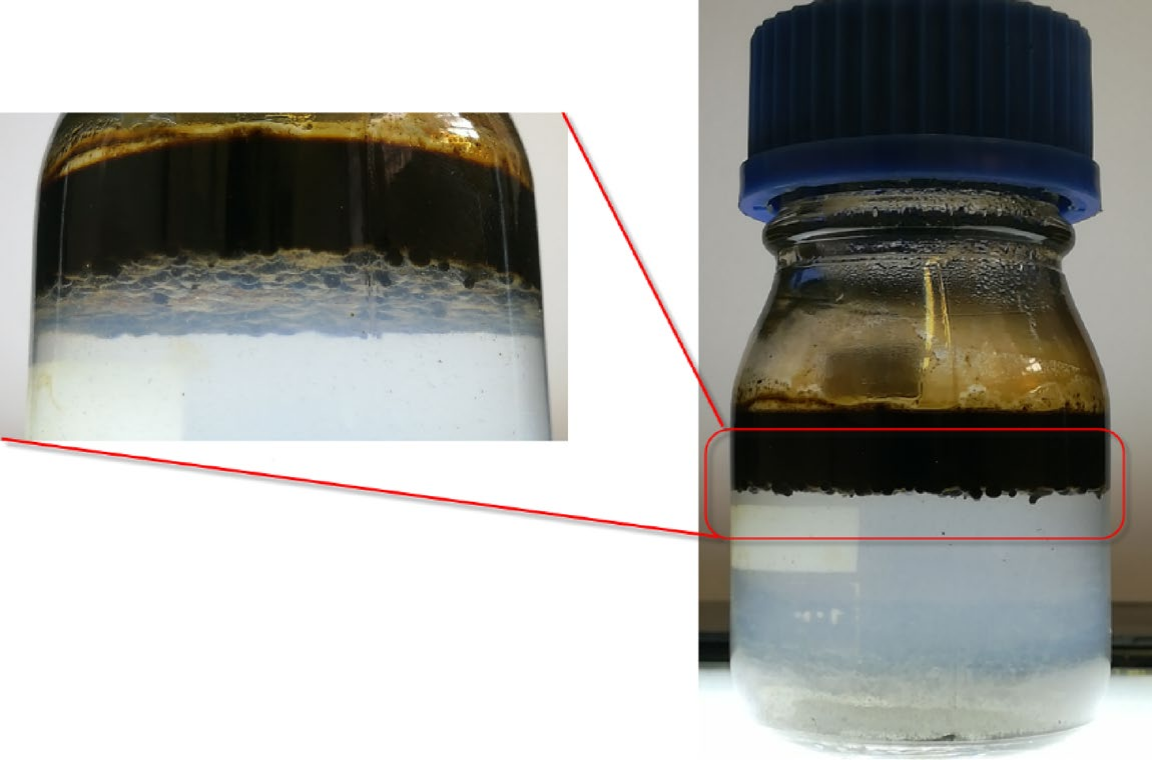
How the synthesized nanostructure is located at the interface between water and crude oil.

In order to check the dual nature (hydrophilic/hydrophobic properties) of the synthesized nanostructure and its placement at the interface of organic matter and water, 0.1% by weight of the synthesized nanostructure with a ratio of 10% of nanotubes to silica was spread in water with the help of an ultrasonic bath, and then the organic matter is added to the sample during stirring sample with the help of a magnetic stirrer. Considering that the color of the synthesized nanostructure is gray to black depending on the ratio of nanotubes to silica and due to the presence of nanotubes, therefore, if crude oil is used as organic material, the presence of the nanostructure at the interface between water and crude oil is not well visible. For this reason, transparent organic materials such as chloroform and engine oil are used to check the placement of the synthesized nanostructure at the interface between water and organic matter. Due to the fact that the density of chloroform is higher than water, chloroform is placed under water. Figure 19 shows the placement of the synthesized nanostructure at the interface of water and organic matters. As can be seen in Fig. 19, the nanostructure is located at the interface of water and engine oil, as well as water and chloroform, which confirms the dual properties of the synthesized nanostructure. The synthesized nanostructures in the samples were still placed on the interface and stable after one month. Figure 20 also shows the location of the synthesized nanostructure at the interface between water and crude oil.

### Investigating the optimal ratio of nanotube to silica

**Fig. 21:**
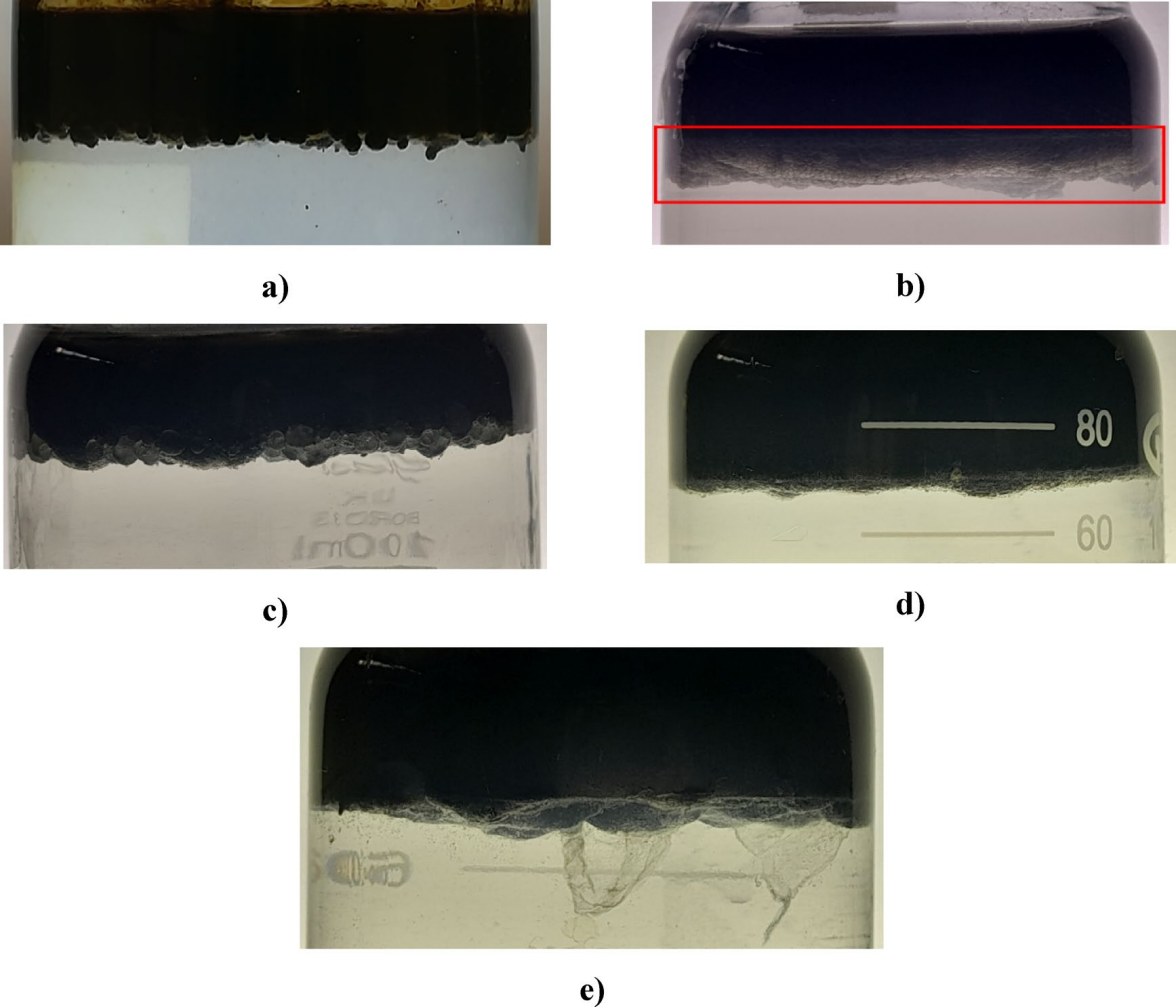
The height of the emulsions between crude oil and water in different ratios of nanotubes to silica, (**a**) 10%, (**b**) 20%, (**c**) 30%, (**d**) 40% and (**e**) 50%.

The emulsion phase formed between water and oil in different ratios of nanotube to silica 10, 20, 30, 40 and 50% is shown in Fig. 21. The interfaces between the three phases are clearly recognizable. In Table 4, the values of the emulsion height and the ratio of the emulsion height to the total mixture height are stated. This ratio shows the emulsification capability of the nanoparticle. As it can be seen, the highest amount of emulsion is formed in the ratio of 20%.

**Table 4:** The ratio of the emulsion height to the total height of the mixture in different ratios of nanotubes to silica.

Ratio of nanotube to silica (%)	Emulsion height (cm)	Total height (cm)	Ratio of the emulsion height to the total height
10	0.3	5	0.06
**20**	**0.6**	**5**	**0.12**
30	0.4	5.1	0.08
40	0.2	5.1	0.04
50	0.1	5.1	0.02

Siginificance value bold.

**Fig. 22:**
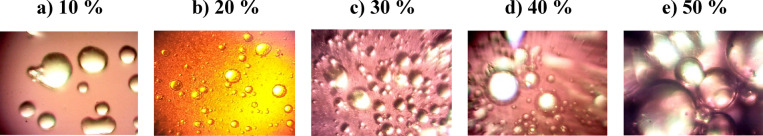
Optical microscope images of the emulsions formed between water and crude oil i in different ratios of nanotubes to silica (the scale of the photos is 10 micrometers).

On the other hand, it is more suitable for the formed emulsion to be more uniform and finer, because it shows that the nanostructure had a better stabilization capability and the surface tension was reduced more. In order to investigate the issue more precisely, the size of the emulsions formed between water and oil in different ratios of nanotubes to silica 10, 20, 30, 40 and 50% were investigated by optical microscope. The results of the recorded images are shown in Fig. 22. It should be noted that due to the change in the light intensity of the microscope for the clarity of the images, the background of the photos has changed, which is inevitable considering that the recorded images were taken at different times. By comparing the images, it can be said that in the ratio of 20, the emulsion droplets have more uniform and smallest size and this ratio causes the lowest surface tension, which confirms the results obtained in the previous part.

As it was observed, the size of the emulsion droplets initially decreases with the increase of nanotubes (or the decrease of silica). But the addition of more nanotubes leads to the opposite effect and the size of the emulsion droplets increase. At this stage, the reduction of silica concentration becomes the dominant factor affecting the droplet diameter. Most importantly, under these conditions, multi-emulsion droplets can be observed (Fig. 22d and e—ratios of 40% and 50%), indicating that low concentrations of hydrophobic particles (silica) may lead to the formation of large droplets and multi-emulsion structures. In other words, excessive increase in hydrophilicity causes instability of the emulsion surface.

One of the important issues in the use of Janus nanostructures is the reuse of these nanoparticle. Filtration techniques can be utilized for Janus nanoparticle recovery. This involves using membranes or filters that can selectively separate the catalysts based on size or other physical properties, ensuring that they can be collected for reuse or further processing^52^. Centrifugation can also be an effective method for separating Janus nanocatalysts from the reaction mixture. By spinning the mixture at high speeds, denser particles (like Janus nanocatalysts) can be separated from lighter components, allowing for their recovery^53^.

## Conclusions

In this study, a new method was proposed for the synthesis of MWCNT-silica hybrid Janus nanostructure. After synthesizing the two- Janus nanostructure by the proposed method, identification and characterization tests were performed on it by FTIR, XRD and TEM tests. By the results of this test, the formation of hybrid Janus nanostructure and its hydrophilicity and oleophilicity were evaluated and its size, shape and morphology were determined. Also, the placement of nanostructures at the interface of water and different organic materials (engine oil, chloroform and crude oil) were investigated. In addition to this, the appropriate ratio of hydrophilicity and oleophilicity was determined by determining the ratio of MWCNT to silica. The following are the conclusions obtained from this study:

1. The results of FTIR test well show the formation of nanotube-silica hybrid. Also, the FTIR test results confirm the hydrophilicity and oleophilicity of the synthesized nanostructure according to the absorption spectrum of carboxylic acid functional groups and alkane and alkene bonds related to oleic acid.
2. Two peaks of graphite (indicating MWCNT) and silica can be recognized from XRD results. Therefore, the matching of the peaks of the synthesized samples with the standard patterns of graphite and silica indicates the formation of hybrid synthesized nanoparticle including MWCNT and silica. The average size of the synthesized crystal was estimated to be about 13 nm using XRD result.
3. TEM is well shown how the silica nanoparticle are attached on the MWCNT structure. Also, with the help of TEM image analysis, the average particle size was estimated to be 14 nm.
4. The combination of the proposed method of masking and mixing for the synthesis resulted in fabrication of the Janus hybrid nanostructure. The synthesized Janus nanostructure is well positioned at the interface of water and engine oil, water and chloroform, as well as water and crude oil, which confirms the dual properties of the synthesized nanostructure.
5. It is more suitable for the formed emulsion to be more uniform and finer, because it shows that the nanostructure had a better stabilization capability and the surface tension was reduced more The results of different weight ratios of nanotube to silica showed that the highest amount of emulsion was formed in the ratio of 20% and the formed emulsions have more uniform and smallest size and this ratio causes the lowest surface tension, therefore Janus nanostructure has a better performance in this ratio.
6. The size of the emulsion droplets initially decreases with the increase of the amount of nanotubes (or the decrease of silica). But the addition of more nanotubes leads to the opposite effect and the size of the emulsion droplets increase.

## Data Availability

All data generated or analysed during this study are included in this article.
